# Quorum Sensing Regulates the Hydrolytic Enzyme Production and Community Composition of Heterotrophic Bacteria in Coastal Waters

**DOI:** 10.3389/fmicb.2021.780759

**Published:** 2021-12-10

**Authors:** Marion Urvoy, Raphaël Lami, Catherine Dreanno, Daniel Delmas, Stéphane L’Helguen, Claire Labry

**Affiliations:** ^1^Ifremer, DYNECO, Plouzané, France; ^2^Université de Bretagne Occidentale, CNRS, IRD, Ifremer, UMR 6539, Laboratoire des Sciences de l’Environnement Marin (LEMAR), Plouzané, France; ^3^Sorbonne Université, CNRS, Laboratoire de Biodiversité et Biotechnologies Microbiennes (LBBM, USR 3579), Observatoire Océanologique de Banyuls, Banyuls-sur-Mer, France; ^4^Ifremer, RDT, Plouzané, France

**Keywords:** hydrolytic enzymes, bacterial community composition, quorum sensing, *N*-acylhomoserine lactone, coastal waters

## Abstract

Heterotrophic microbial communities play a central role in biogeochemical cycles in the ocean by degrading organic matter through the synthesis of extracellular hydrolytic enzymes. Their hydrolysis rates result from the community’s genomic potential and the differential expression of this genomic potential. Cell-cell communication pathways such as quorum sensing (QS) could impact both aspects and, consequently, structure marine ecosystem functioning. However, the role of QS communications in complex natural assemblages remains largely unknown. In this study, we investigated whether *N*-acylhomoserine lactones (AHLs), a type of QS signal, could regulate both hydrolytic activities and the bacterial community composition (BCC) of marine planktonic assemblages. To this extent, we carried out two microcosm experiments, adding five different AHLs to bacterial communities sampled in coastal waters (during early and peak bloom) and monitoring their impact on enzymatic activities and diversity over 48 h. Several specific enzymatic activities were impacted during both experiments, as early as 6 h after the AHL amendments. The BCC was also significantly impacted by the treatments after 48 h, and correlated with the expression of the hydrolytic activities, suggesting that changes in hydrolytic intensities may drive changes in BCC. Overall, our results suggest that QS communication could participate in structuring both the function and diversity of marine bacterial communities.

## Introduction

Heterotrophic bacterial communities play a central role in carbon (C) and nutrient cycling in the oceans (Azam and Malfatti, 2007; Falkowski et al., 2008). They rely on the expression of a large diversity of dissolved and cell-bound extracellular enzymes to hydrolyze polymeric organic matter into smaller molecules that can be transported into the cells and metabolized (Payne, 1980; Arnosti, 2011). Hydrolytic enzymes initiate the mineralization of organic matter, ultimately affecting key biogeochemical processes such as C export or nutrient cycling (Chróst, 1990; Azam and Malfatti, 2007). As such, it is essential to understand the mechanisms driving hydrolase expression in marine bacterial communities.

Hydrolytic activity levels result from a combination of genomic potential, determined by the bacterial community composition (BCC), and the differential expression of this genetic potential (Arnosti, 2011). Indeed, bacterial communities may possess different degradation pathways, depending on the presence of specific taxonomic groups (Zimmerman et al., 2013; Arnosti, 2014), resulting in different hydrolytic enzyme capacities (Arnosti, 2011). At the transcriptional level, several factors may drive enzyme synthesis, such as the composition and concentration of organic matter or the nutrient levels (Chróst, 1990; Arnosti, 2011). However, despite their broad biogeochemical implications, we still lack knowledge about the factors and mechanisms driving bacterial extracellular enzyme production (Arnosti, 2011, 2014) and BCC (Konopka et al., 2015) in marine environments, especially concerning the role of biotic interactions (Arnosti, 2014; Cosetta and Wolfe, 2019).

Quorum sensing (QS) is a bacterial signaling system allowing the synchronized expression of numerous genes among a bacterial population (Miller and Bassler, 2001; Waters and Bassler, 2005). QS communications are based on the production, diffusion, and sensing of diffusible molecules called autoinducers (AIs). When the AI concentration reaches a certain threshold, they bind their cognate receptors and initiate the transcriptional regulation of their target genes (Fuqua et al., 1994; Papenfort and Bassler, 2016), including genes involved in bioluminescence, motility, biofilm formation, virulence, or hydrolytic enzyme production (Miller and Bassler, 2001; Hmelo, 2017). Quorum quenching (QQ) encompasses mechanisms that either degrade AI molecules or impede their production or reception (Grandclément et al., 2015). In gram-negative bacteria, the most studied QS system is based on the expression of *N*-acylhomoserine lactones (AHLs), consisting of a homoserine lactone and a fatty acid side-chain (Papenfort and Bassler, 2016; Hmelo, 2017). AHL-based QS is widespread among marine bacteria: diverse QS genes have been found during metagenomic surveys (Doberva et al., 2015; Muras et al., 2018; Su et al., 2021), and AHLs have been detected in a few environments such as marine aggregates (Hmelo et al., 2011; Jatt et al., 2015), subtidal biofilms (Huang et al., 2009), or intertidal sediment (Stock et al., 2021). In addition, numerous strains isolated from marine environments produced AHLs during cultivation (Gram et al., 2002; Blanchet et al., 2017; Su et al., 2019; Urvoy et al., 2021).

However, the implication of QS expression on key biogeochemical functions is largely unknown and mostly stems from cultivation-dependent methods (Lami, 2019), which lack the inherent complexity of natural bacterial assemblages. QS expression could substantially impact functional phenotypes through direct transcriptional regulation of hydrolytic enzymes and through the modification of BCC. Indeed, QS regulation of hydrolytic enzymes at the transcriptional level has been demonstrated in several environmental strains (Jatt et al., 2015; Su et al., 2019; Urvoy et al., 2021). A limited number of studies have investigated the link between QS and hydrolytic activities in natural communities (Hmelo et al., 2011; Van Mooy et al., 2012; Krupke et al., 2016; Su et al., 2019). Hmelo et al. (2011) were the first to suggest that the expression of QS promoted the degradation of particulate organic matter, demonstrating that the addition of AHL to bacterial communities colonizing marine snow increased the activity of several hydrolases. This finding was later supported by Krupke et al. (2016) and Su et al. (2019), who performed similar experiments on marine snow, extending the impact of QS-regulation to other enzymatic activities and locations. Krupke et al. (2016) highlighted the complexity of those cell-cell mechanisms, as the responses varied depending on the sampling location, AHL concentration, and time scale. Additionally, Van Mooy et al. (2012) broadened those observations to the epibiont of *Trichodesmium* colonies, where QS seemed to regulate phosphorus acquisition through alkaline phosphatases regulation.

Even fewer studies have looked into the interplay between QS and BCC in marine communities so far. Huang et al. (2019) demonstrated that AHL-based QS disruption modified the composition of marine communities colonizing steel coupons. Whalen et al. (2019) showed that alkylquinolone signals, another type of AIs, contributed to the structuration of both free-living and particle-attached bacterial communities during a simulated coastal phytoplankton bloom. In other environments, Schwab et al. (2019) demonstrated that AHL signal disruption affected the BCC in both biofilm-forming and suspended soil bacterial communities. Studies performed on sludge (Gao et al., 2018; Lv et al., 2018; Ma et al., 2018) or a bio-membrane reactor (Jo et al., 2016) also pointed out that AHL amendment or AHL disruption modulated both BCC and bacterial metabolism rates. However, to the best of our knowledge, no studies have shown the direct influence of AHL-based QS on the BCC in the marine environment, and the ecological role of QS remains understudied.

In this study, we investigated the influence of QS on hydrolytic enzyme production and taxonomic diversity of marine heterotrophic prokaryotic communities. To this extent, two independent microcosm-based experiments were performed, where five different AHLs were separately amended to natural assemblages from the Bay of Brest (France). Both hydrolytic potential and BCC were monitored over 48 h. Altogether, our results suggest that QS is involved in the regulation of several hydrolytic enzymes and may influence the composition of marine bacterial communities.

## Materials and Methods

### Chemical Reagents and *N*-Acylhomoserine Lactone Preparation

The AHLs used were purchased from Sigma-Aldrich (Darmstadt, Germany) or Cayman Chemical (Ann Arbor, MI, United States): *N*-butanoyl-DL-homoserine lactone (C4-HSL), *N*-hexanoyl-DL-homoserine lactone (C6-HSL), *N*-(3-Oxooctanoyl)-DL-homoserine lactone (3-oxo-C8-HSL), *N*-dodecanoyl-DL-homoserine lactone (C12-HSL), *N*-tetra decanoyl-DL-homoserine lactone (C14-HSL), *N*-hexadecanoyl-DL-homoserine lactone (C16-HSL). All AHLs were dissolved in dimethyl sulfoxide (DMSO) at 50 μM, stored at −20°C, and thawed at the beginning of the experiment. All other chemicals were purchased from Sigma-Aldrich, Darmstadt, Germany (biochemical grade).

### Effect of *N*-Acylhomoserine Lactone Amendment on Functional and Taxonomic Diversity

Two independent microcosm experiments were performed to assess the variability in response to AHL amendments. To this extent, seawater was sampled near the Service d’Observation en Milieu LITtoral (SOMLIT)^1^ station of Sainte-Anne-du-Portzic (48°21′33.5″ N, 4°33′02.7″ W, Bay of Brest, France) on March 29 (10.7°C *in situ*, salinity 32.4) and May 3, 2021 (12.3°C, salinity 33.7), using acid-washed 10-L carboys (Nalgene). The two sampling dates correspond to two contrasting periods, namely the beginning (high nutrient, low chlorophyll *a* levels) and the peak (low nutrient, high chlorophyll *a* levels) of the annual phytoplankton spring growth (Supplementary Figure 1). Additional details concerning the bloom’s characteristics are given in Supplementary Figure 1. The sampled seawater was immediately 10-μm-filtered (Merck, Ref NY1004700) to remove aggregates and larger eukaryotic cells, stored at *in situ* temperature, and processed within 1 h. The bacterial community is later referred to as planktonic because of the removal of bacteria attached to those aggregates.

**FIGURE 1:**
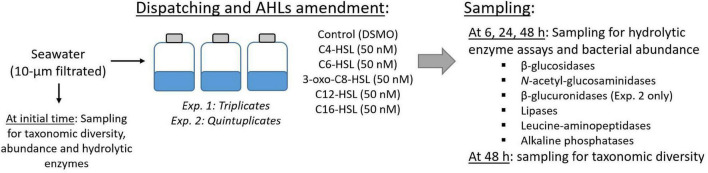
Design of the study. Seawater was sampled on two separated occasions (early and peak bloom) and 10-μm-filtrated to remove aggregates and larger eukaryotes. The seawater was then dispatched, and 50 nM of AHLs or DMSO were amended. The hydrolytic enzyme activities were assayed at 0, 6, 24, and 48 h. Initial and final diversity were assessed using partial 16S rDNA gene metabarcoding. Exp., experiment.

To assess the effect of AHLs on hydrolytic enzymes and taxonomic diversity, 150 mL of the 10-μm filtered seawater was dispatched into T-175 flasks (Sarstedt cat. no. 83.3912), and five different AHLs (C4-, C6-, 3-oxo- C8-, C12-, and C16-HSL) were separately amended at a final concentration of 50 nM (0.1% v/v of DMSO final concentration). The different AHLs were chosen to cover a large spectrum of AHL side-chain lengths. The AHL concentration (50 nM) was chosen based on previous results (Supplementary Figure 2), aiming to maximize the enzymatic activity response and minimize the AHL concentration to limit their use as a nutritional source. In addition, a control condition was prepared with 0.1% (v/v) DMSO. The incubations were performed at *in situ* temperature in the dark, in triplicates (First experiment) or in quintuplicates (Second experiment). Samples for hydrolytic enzyme activities and bacterial abundance were collected in sterile tubes (Falcon, Ref 352070) at 0 (on initial water before dispatching), 6, 24, and 48 h. The initial BCC was determined in triplicate by filtering 200 mL of 10-μm filtered seawater (before dispatching) through 0.2-μm filters (Whatman Nuclepore PC membrane). The final diversity was sampled by filtering the leftover seawater in each microcosm (approximately 100 mL) through 0.2-μm filters. The experimental design is schematized in Figure 1.

### Bacterial and Phytoplankton Abundance Measurement

Bacterial and phytoplankton abundances were quantified according to standard flow cytometry protocols (Marie et al., 1999). Bacterial samples were fixed with 0.25% glutaraldehyde and 0.01% Poloxamer 188 for 10 min at ambient temperature in the dark. Samples were then flash-frozen in liquid nitrogen and stored at −80°C until flow cytometry analysis. Samples were thawed, appropriately diluted in 0.2-μm-filtered autoclaved seawater, and labeled with SYBR Green I (1:10,000 dilution of stock solution) for 10 min at ambient temperature in the dark. The acquisition was performed on a Novocyte Advanteon Cytometer with a threshold on FITC, recording less than 1,000 events per second. Bacteria were discriminated using the green and orange fluorescence of SYBR Green I and forward and side scatter criteria. For each microcosm, abundance was averaged over triplicated samples.

In addition, the initial phytoplankton cell counts were measured using the same samples, at the initial time (10-μm filtered seawater before dispatching) and in the DMSO-treated microcosms at the final time (48 h). To this extent, the cytometry acquisition was performed using 250 μL of undiluted, unlabeled samples. The picophytoplankton, nanophytoplankton, cryptophytes and cyanobacteria cells were discriminated using the orange and red fluorescence. This measurement suggested that phytoplankton cells were correctly removed from the microcosms (Supplementary Figure 3).

### Hydrolytic Enzyme Assay

Several hydrolytic enzymes involved in different biogeochemical cycles were assayed: β-glucosidases (β-glc), β-glucuronidases (β-glucu), and *N*-acetyl-glucosaminidases (N-ac) degrade polysaccharides (C and N cycles), lipases (Lip) degrade lipids (C cycle), leucine aminopeptidases (LAM) degrade peptides (N and C cycles), and alkaline phosphatases (AP) are used to acquire phosphorus (P). Activities were determined using model fluorogenic substrates which release either 7-amido-4-methylcoumarin (MCA) or 4-Methylumbelliferone (MUF) upon hydrolysis. Those substrates were, respectively: 4-Methylumbelliferyl-β-D-glucopyranoside (MUF-β-glc), 4-Methylumbelliferyl-β-D-glucuronide (MUF-β-glucu), 4-Methylumbelliferyl N-acetyl-β-D-glucosaminide (MUF-N-Ac), 4-Methylumbelliferyl butyrate (MUF-but), L-Leucine-7-amido-4-methylcoumarin (LLMCA), and 4-Methylumbelliferyl phosphate (MUF-P). Substrates and standards for enzymatic assays were dissolved in 2-methoxyethanol using sonication if necessary, and stored at −20°C.

Total potential activities were assayed, which include dissolved and cell-bound enzymes. To this extent, 80 μL of sample was dispatched in a 384-well low-binding black microplate (Greiner Item no. 781900) and amended with 20 μL of Tris buffer (10 mM, pH 8.2) containing the fluorogenic substrate at saturating concentration. Saturating concentrations were previously determined as follows: 150 μM MUF-β-glc, 250 μM MUF-β-glucu, 250 μM MUF-N-ac, 800 μM MUF-but, 1,000 μM LLMCA, and 250 μM MUF-P (data not shown). Tris buffer was used to avoid bias induced by pH changes over time as MUF and MCA fluorescence yields are highly pH-dependent. Additionally, a control for substrate abiotic degradation was prepared with 80 μL of Tris buffer instead of seawater sample. Given the number of samples in the Second experiment, the activities were measured in two separate microplates: LAM, AP, and Lip were measured first, followed by β-glc, β-glucu, and N-ac. To avoid any bias, fresh samples were collected in new tubes before the second incubation.

Fluorescence was monitored every 11 min for 3–4 h using a Spark Tecan Infinite M200PRO with excitation/emission wavelengths of 364/460 nm for MUF and 380/440 nm for MCA. Activities were then determined as the slope of the linear part of the curve (AU min^–1^). Blanks corresponding to abiotic degradation were subtracted from the samples. Only MUF-but presented a substantial abiotic degradation. Fluorescence units were converted into substrate equivalents using a standard curve of MCA and MUF dissolved in 20 μL of Tris buffer and 80 μL of 0.2-μm-filtered and autoclaved seawater. Enzymatic activities were determined as the average of technical duplicates (First experiment) or quadruplicates (Second experiment). Specific activities were obtained by normalizing activities with bacterial abundance and expressed as amol_*MUF or*_ _*MCA*_ h^–1^ 10^6^ cells^–1^.

### Bacterial Taxonomic Diversity

The BCC was determined by metabarcoding of the V3/V4 region of the 16S rRNA gene. Filters dedicated to bacterial diversity were immediately flash-frozen in liquid nitrogen and stored at −80°C until processing. Blank dry filters were sampled simultaneously and used as a contamination control. The two experiments were processed simultaneously. Filters were cut into pieces and DNA was extracted using the NucleoSpin Plant II Mini Kit (Macherey Nagel Ref. 740770.50) according to the manufacturer’s instructions, with an additional lysis step performed for 2 h at 56°C with 25 μL of proteinase K (20 mg mL^–1^, Macherey Nagel Ref. 740506) and 100 μL of lysozyme (20 mg mL^–1^, Sigma ref 4403-5g). Libraries were prepared and sequenced by Génome Québec using the 341F/785R primers (Klindworth et al., 2013). Samples were sequenced on an Illumina MySeq using 2 × 300 pb and V3 chemistry. Data were processed using the SAMBA pipeline (v3.0.1)^2^ developed by the IFREMER bioinformatics team (SeBiMER). This resulted in 2,120 amplicon sequence variants (ASVs), which were then clustered using dbOTU3 (Olesen et al., 2017), resulting in 1,502 ASVs (29% clustering) that were assigned against the Silva v138 database (Quast et al., 2013).

### Statistical Analysis

All data were analyzed in R (v4.0.3, 2020-10-10) and displayed using ggplot2 (v3.3.2). Plots representing abundance and specific enzymatic activity display biological replicates as dots and means and standards errors as crossbars. Statistical comparison of the specific enzymatic activity in the different treatments was performed using the *t*-test (function *compare_means*, ggpubr package v0.4.0) with the Benjamini-Hochberg correction for *p*-values (*p*_*BH*_). Results were plotted using compact letter display as implemented in the rcompanion package (v2.3.26), applying a significance threshold of 0.1.

Metabarcoding data were analyzed in R using the Phyloseq (v1.32.0) and Vegan (v2.5.7) packages. The ASVs corresponding to eukaryotes, mitochondria, and chloroplasts were removed (6.4% of reads). The barplots representing the relative abundances of the ASVs were plotted based on the raw count table transformed to relative abundance. The rarefaction curves (Supplementary Figure 4) were visualized using the *ggrare* function from the ranacapa package (v0.1.0). Data were then rarefied to the minimum sampling depth (32,196 sequences per samples) (*rarefy_even_depth* function, rngseed = 999). Principal Coordinates Analysis (PCoA) was performed on the rarefied table, based on the Bray-Curtis dissimilarity. Permutational multivariate analysis of variance (PERMANOVA) was done on the rarefied table using the *adonis* function (999 permutations) based on the Bray-Curtis dissimilarity, using the treatment as a grouping variable (DMSO, C4-, C6-, 3-oxo- C8-, C12-, and C16-HSL). Homogeneity of variance was checked using the *betadisper* function. Analysis of similarities (ANOSIM) was performed on the rarefied table using the *anosim* function (999 permutations) based on Bray-Curtis dissimilarity, using the same groups. The DESeq2 package (v1.28.1) was used on the raw count table to detect ASVs that were differentially abundant between the AHL treatments (C4-, C6-, 3-oxo- C8-, C12-, and C16-HSL) and the DMSO control in each experiment. Low-prevalence ASVs (five reads or less across one experiment) were removed beforehand. The cut-offs used to consider the DESeq2 results significant were as follows: *p*_*BH*_ < 0.05 and log2 fold change (log2FC) > 0.5 or < −0.5.

The association between the specific enzymatic activities and the BCC at the end of the incubation was assessed using a symmetric Procrustes analysis. This analysis aimed to test if the distance matrix of the community shows superposition with the distance matrix of the activities. To this extent, the rarefied community matrix was Hellinger-transformed (function *decostand*) and ordinated using a Principal Components Analysis (PCA, as implemented in the *rda* function in the Vegan package). The specific activities (at 48 h) were scaled to unit variance and ordinated using a PCA (*rda* function). Symmetric Procrustes analysis was performed on the two resulting ordinations (function *procrustes* in the Vegan package) and its significance was assessed (function *protest* in the Vegan package) using 999 permutations.

## Results

### Effect of *N*-Acylhomoserine Lactone Amendment on Bacterial Abundance

**FIGURE 2:**
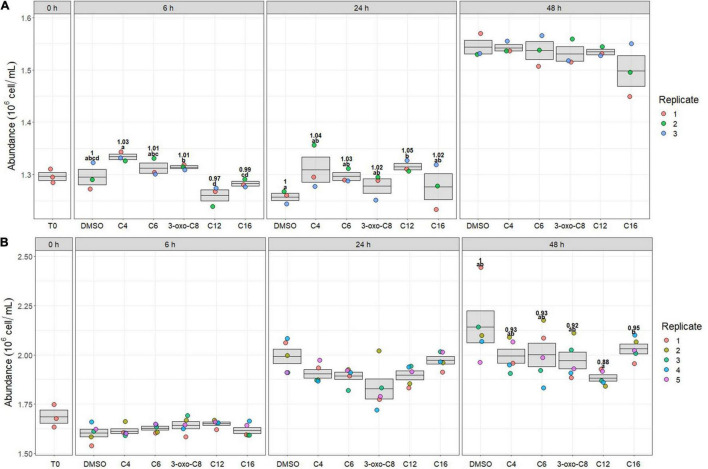
Bacterial abundances at 0, 6, 24, and 48 h for the first **(A)** and second **(B)** experiment, corresponding to early and peak bloom conditions, respectively. Significance of the mean abundance comparison between treatments is indicated using compact letter display (*t*-test, *p*_*BH*_ < 0.1). Ratio of the mean abundance compared to DMSO treatment is annotated for each time with significantly different results.

During the First experiment (early bloom), bacterial growth was slow and mainly occurred between 24 and 48 h (Figure 2A). Mean bacterial abundance increased from 1.30 to 1.53 × 10^6^ cells mL^–1^ (+18%) between 0 and 48 h. Overall, bacterial growth was not significantly different between the DMSO and the AHL-treated microcosms, except at 24 h, where the abundance was increased in the C12-HSL microcosms (+5%, *p*_*BH*_ = 0.06). Significant differences were also observed between the AHL-treated microcosms at 6 h, as the C4-HSL-treated microcosms contained more bacteria than the microcosms treated with 3-oxo- C8-, C12-, and C16-HSL (largest increase of +6% between C4- and C12-HSL, *p*_*BH*_ = 0.05).

During the Second experiment (peak bloom), bacterial growth was also slow and started between 6 and 24 h (Figure 2B). Mean bacterial abundance increased from 1.69 to 2.00 × 10^6^ cells mL^–1^ (+18%) between 0 and 48 h. Bacterial growth was not significantly different between the DMSO control and the AHL-treated microcosms, although bacteria were significantly less abundant in the C12-HSL-treated microcosms than in the C16-HSL-treated microcosms at 48 h (−7%, *p*_*BH*_ = 0.02).

### Effect of *N*-Acylhomoserine Lactone Amendment on Hydrolytic Enzyme Activities

**FIGURE 3:**
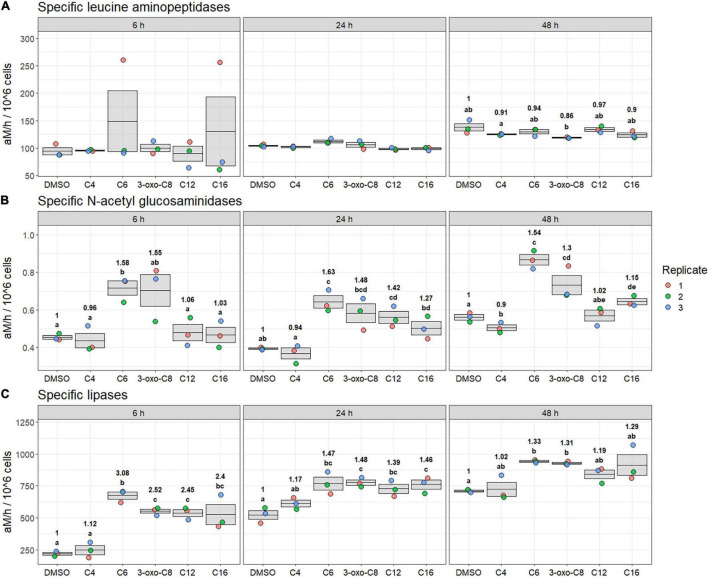
Specific leucine-aminopeptidase **(A)**, N-acetyl-glucosaminidases **(B)**, and lipase **(C)** activities at 6, 24, and 48 h during the first experiment (early bloom). Significance of the mean specific activity comparison between treatments is indicated using compact letter display (*t*-test, *p*_*BH*_ < 0.1). Ratio of the mean specific activity compared to DMSO treatment is annotated for each time with significantly different results.

During the First experiment (early bloom), AHL amendment affected specific LAM, N-ac, and Lip activities (Figure 3). Although no AHL-treated microcosms were significantly different from the control condition, specific LAM activities were affected by the treatment at 48 h: LAM activities in the 3-oxo-C8-HSL-treated microcosm were significantly reduced compared with those in the C4-HSL-treated microcosm (−5%, *p*_*BH*_ = 0.10, Figure 3A).

The AHL effect on specific N-ac activity was visible at all sampling times (Figure 3B). Overall, the microcosms containing the C6-HSL were the most affected ones: the treatment induced an average +58% increase in activity compared with DMSO treatment across all sampling times (*p*_*BH*_ < 0.08). The microcosms containing 3-oxo-C8-HSL followed a similar pattern, although the induction was only significant at 48 h (+30%, *p*_*BH*_ = 0.10). The AHLs with longer side-chains (C12- and C16-HSL) also increased the specific N-ac activity, albeit to a lesser extent: C12-HSL significantly induced the activity at 24 h (+42%, *p*_*BH*_ = 0.09), whereas C16-HSL significantly induced the activity at 48 h (+15%, *p*_*BH*_ = 0.05). On the contrary, C4-HSL-amended microcosms exhibited a decrease in activity at 48 h (−10% compared with the DMSO control, *p*_*BH*_ = 0.10).

The AHL amendment also affected specific Lip activities at all sampling times (Figure 3C). At 6 and 24 h, Lip activities were induced in all AHL-containing microcosms compared with the DMSO treatment, except for the C4-HSL treatment which did not differ from the control. At 6 h, C6-HSL had the most effect (+208%, *p*_*BH*_ = 0.01). The 3-oxo- C8-, C12-, and C16-HSL had a similar effect at this sampling time, with an average +146% increase in specific activity (*p*_*BH*_ < 0.08). For those four AHLs (C6-, 3-oxo- C8-, C12-, and C16-HSL), the treatment effect then lessened with time: at 24 h, the activity was significantly increased by an average of +39% (*p*_*BH*_ < 0.06). At 48 h, the induction was only significant for the C6- and 3-oxo-C8-HSL (+33 and +31%, respectively, *p*_*BH*_ < 0.001).

Finally, no significant effect of AHL amendment was observed on β-glc, AP were too low to be detected, and β-glucu were not measured during the First experiment (data not shown).

**FIGURE 4:**
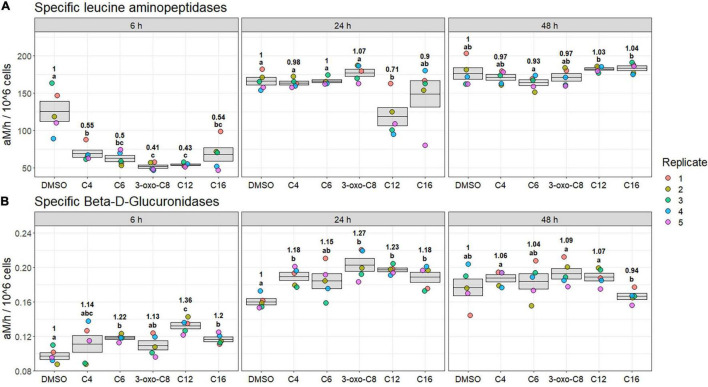
Specific leucine-aminopeptidase **(A)** and β-glucuronidase **(B)** activities at 6, 24, and 48 h during the second experiment (peak bloom). Significance of the mean specific activity comparison between treatments is indicated using compact letter display (*t*-test, *p*_*BH*_ < 0.1). Ratio of the mean specific activity compared to DMSO treatment is annotated for each time with significantly different results.

During the Second experiment (peak bloom), there was an effect of AHL addition on specific LAM and β-glucu activities (Figure 4). At 6 h, all AHL amendments had led to a reduction of LAM activity, with a decrease ranging from −45 to −59% (*p*_*BH*_ = 0.03, Figure 4A). Those effects were especially pronounced for 3-oxo-C8- and C12-HSL amendments (−59 and −57%, respectively, *p*_*BH*_ = 0.03). The AHL impact then lessened over time: at 24 h, all microcosms had reverted to the control level, except for C12-HSL-amended microcosms (−29%, *p*_*BH*_ = 0.07). At 48 h, no microcosm was significantly different from the control, although in C12- and C16-HSL microcosms, activity levels were significantly higher than in C6-HSL microcosms (+11% on average, *p*_*BH*_ = 0.05).

Significant effects were also observed on specific β-glucu activity (Figure 4B). At 6 h, β-glucu activity in the C6-, C12-, and C16-HSL-amended microcosms was significantly induced compared with the control condition (+14 to +36%, *p*_*BH*_ < 0.02), with the most pronounced effect resulting from C12-HSL treatment (+36%, *p*_*BH*_ = 0.003). At 24 h, there was a +18 to +27% activity increase in the C4-, 3-oxo-C8, C12-, and C16-HSL-amended microcosms compared with the control (*p*_*BH*_ < 0.01). The activity in the C6-HSL-amended communities also seemed induced, although not significantly. At 48 h, all microcosms were similar to the control condition, but the activity in the C16-HSL microcosms was significantly lower than in the C4-, 3-oxo- C8-, and C12-HSL-treated microcosms (*p*_*BH*_ < 0.04).

In addition, no significant effect was observed on β-glc, AP, and N-ac (data not shown). Finally, the Lip measurements were discarded as the abiotic blank had a similar activity level than the samples, resulting in negative values. In any case, AHL-amendment had a limited effect on this activity: none of the AHL-treated microcosms differed from the DMSO control, although C12- and C16-HSL were significantly higher than C4-, C6-, and 3-oxo-C8-HSL at 24 h (*p*_*BH*_ < 0.08, data not shown).

### Effect of *N*-Acylhomoserine Lactone Amendment on Taxonomic Diversity

**FIGURE 5:**
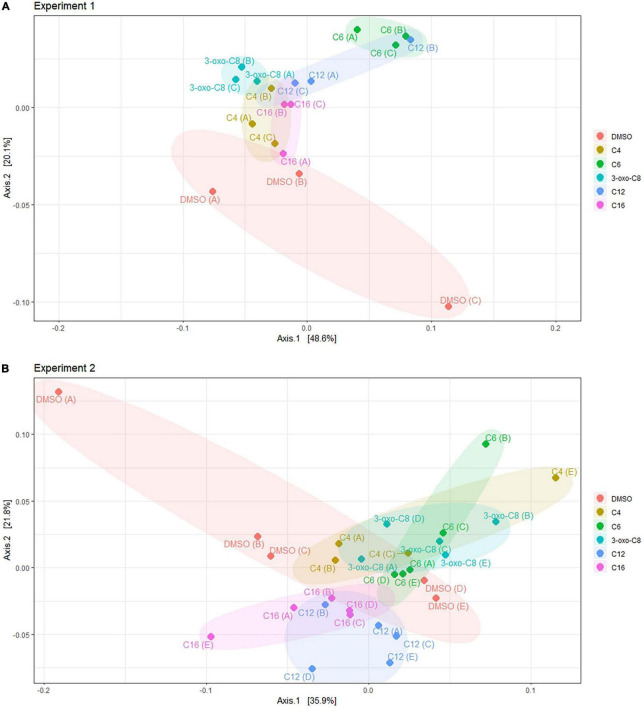
PCoA ordination based on the Bray-Curtis dissimilarity, at the end of the incubation for the First **(A)** and Second **(B)** experiment, corresponding to early and peak bloom conditions, respectively. Ellipses were drawn to facilitate visualization but do not hold a statistical meaning.

The effect of the AHL treatment on the BCC at the end of the First experiment (early bloom) was visualized using PCoA (Figure 5A), explaining 68% of the variability. This analysis separated the AHL-treated communities from the DMSO control communities. The C6-HSL-treated communities were the furthest from the control communities, followed by the 3-oxo-C8- and C12-HSL-treated communities. The PERMANOVA and ANOSIM tests both supported a significant effect of the AHL-treatment (PERMANOVA: *p* = 0.002, *R*^2^ = 0.52, *F*-statistic = 2.60; ANOSIM: *p* = 0.001, *R*-statistic = 0.45), although both *F*- and *R*-statistics suggested a limited size-effect. The test for the homogeneity of variance did not show a significant difference in variance among the treatments (*p* = 0.46), supporting the PERMANOVA results.

**FIGURE 6:**
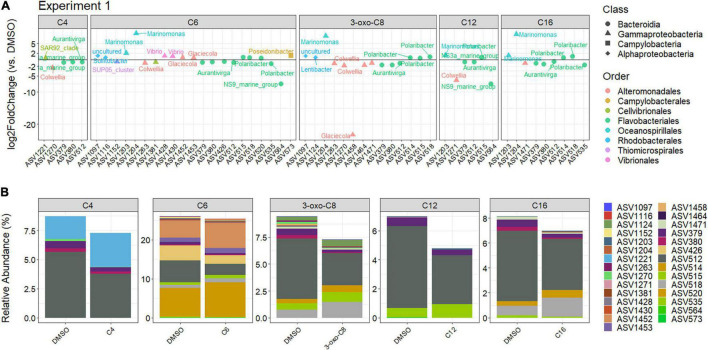
**(A)** Log2 fold change of the differentially abundant ASVs for each AHL-treatment compared to the DMSO control during the First experiment, corresponding to the beginning of the bloom (cut-off *p*_*BH*_ = 0.05, cut-off log2FC = 0.5 or –0.5). **(B)** Relative abundances of the differentially abundant ASVs for each treatment compared to the DMSO control during the First experiment.

Differential abundance analysis for the First experiment highlighted 29 ASVs (out of 497 ASVs, i.e., 5.8%) that were significantly different in at least one AHL treatment compared to the DMSO control (Figure 6A). Coherently with the PCoA, C6-HSL treatment had the most effect on the BCC with 21 impacted ASVs, including 9 ASVs affiliated with Bacteroidia (all belonging to the Flavobacteriales order), 9 affiliated with Gammaproteobacteria (3 Alteromonadales, 2 Vibrionales, 2 Oceanospirillales, 1 Cellvibrionales, and 1 Thiomicrospirales), 2 affiliated with Alphaproteobacteria (2 Rhodobacterales), and 1 affiliated with Campylobacteria (Campylobacterales). Exposure to C6-HSL increased the relative abundance of Rhodobacterales, Oceanospirillales, Vibrionales, and Campylobacterales but decreased those of Thiomicrospirales (Gammaproteobacteria) and Cellvibrionales. The effect on Flavobacteriales and Alteromonadales was more contrasted with both increase and decrease in relative abundances. The 3-oxo-C8-HSL was the second-most potent AHL, affecting 14 ASVs (6 Bacteroidia, 2 Alphaproteobacteria, and 6 Gammaproteobacteria). The 3-oxo-C8-HSL increased the abundance of the Rhodobacterales, Oceanospirillales, and three Flavobacteriales (*Polaribacter* and *Aurantivirga* ASVs) but decreased the relative abundance of Alteromonadales and two Flavobacteriales (*NS3a marine group*). The C4-, C12-, and C16-HSL treatment affected five ASVs (3 Bacteroidia and 2 Gammaproteobacteria), six ASVs (4 Bacteroidia and 2 Gammaproteobacteria), and nine ASVs (6 Bacteroidia and 3 Gammaproteobacteria), respectively, with both increase and decrease in relative abundances. Interestingly, 14 of those 29 differentially abundant ASVs were affected by at least two AHLs (ASVs 379, 380, 512, 514, 515, 518, 535, 564, 1,097, 1,203, 1,204, 1,263, 1,270, and 1,471, Supplementary Figure 5, top), mostly belonging to the Flavobacteriales order. Among them, ASVs 379 and 512, two abundant Flavobacteriales (Figure 6B and Supplementary Table 1), were affected by all AHLs with a similar impact (respective mean log2FC of −1.00 and −0.73).

Although few ASVs were differentially abundant across the AHL treatments (29 out of 497 ASVs), 13 (45%) of them (ASV 379, 380, 426, 512, 514, 515, 518, 520, 1,124, 1,152, 1,221, 1,452, and 1,453) were in the 50 most abundant ASVs present in the DMSO control at the end of the incubation (Figure 6B and Supplementary Table 1), representing 27.7% of the control community.

At the end of Second experiment (peak bloom), PCoA (Figure 5B, 55% of variance explained) indicated a separation between the communities amended with short-chain AHLs (C4-, C6-, and 3-oxo-C8-HSL) and those amended with long-chain AHLs (C12- and C16-HSL). Similar to the First experiment, the PERMANOVA and ANOSIM results both supported a significant effect of the treatment, with a small size-effect (PERMANOVA: *p* = 0.001, *R*^2^ = 0.35, *F*-statistic = 2.60; ANOSIM: *p* = 0.001, *R*-statistic = 0.29) and the test for homogeneity of variance did not show a significant difference in variance (*p* = 0.36).

**FIGURE 7:**
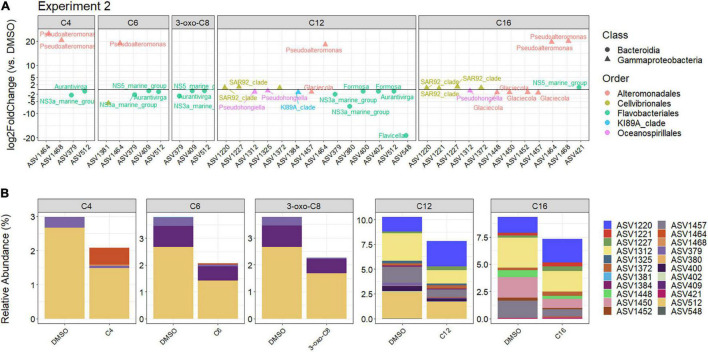
**(A)** Log2 fold change of the differentially abundant ASVs for each AHL-treatment compared to the DMSO control during the Second experiment, corresponding to the peak of the bloom (cut-off *p*_*BH*_ = 0.05, cut-off log2FC = 0.5 or –0.5). **(B)** Relative abundances of the differentially abundant ASVs for each treatment compared to the DMSO control during the Second experiment.

Differential abundance analysis highlighted a total of 22 ASVs (out of 450 ASVs i.e., 4.9%) that were significantly different in at least one AHL treatment (Figure 7A) at the end of the Second experiment. Coherently with the PCoA results, the effect of the AHL treatment on the differential abundance of ASVs seemed to differ according to the side-chain length of the AHL (Figures 7A,B). The C4-, C6-, and 3-oxo-8-HSL affected four ASVs (2 Bacteroidia and 2 Gammaproteobacteria), five ASVs (3 Bacteroidia and 2 Gammaproteobacteria), and three ASVs (3 Bacteroidia), respectively. The ASV 379 and 512 (2 Flavobacteriales) were commonly affected by the three AHLs, which induced similar effects (Figure 7 and Supplementary Figure 5). Interestingly, those three AHLs reduced the relative abundances of the Flavobacterial-affiliated ASVs (log2FC between −2.77 and −0.50) but increased the relative abundance of the *Pseudoalteromonas*-affiliated ASVs (log2FC > 19). The C12-HSL treatment had the most impact, with 14 affected ASVs, 6 being affiliated with Bacteroidia (all Flavobacteriales, including the ASV 379 and 512, also affected by the shorter-chain AHLs) and 8 with Gammaproteobacteria (3 Cellvibrionales, 2 Alteromonadales, 2 Oceanospirillales, and 1 KI89A clade). The C12-HSL reduced the relative abundances of all Flavobacterial–affiliated ASVs. The effect on Gammaproteobacteria was more contrasted, with a relative increase in Cellvibrionales and *Pseudoalteromonas* (Alteromonadales) but a decrease in Oceanospirillales, KI89A clade and *Glaciecola* (Alteromonadales). The C16-HSL affected 1 Bacteroidia (Flavobacteriales) and 11 Gammaproteobacteria ASVs (6 Alteromonadales, 4 Cellvibrionales, and 1 Oceanospirillales), with six ASVs being in common with the C12-HSL treatment. The C16-HSL effect was similar to the C12-HSL since it increased the relative abundances of Cellvibrionales and *Pseudoalteromonas* but decreased those of Oceanospirillales and *Glaciecola*. Interestingly, 10 of those 22 differentially abundant ASVs were affected by at least two AHLs (ASVs 379, 409, 512, 1,220, 1,227, 1,312, 1,372, 1,457, 1,464, and 1,468, Supplementary Figure 5, bottom). The ASVs 379 and 512 (Flavobacteriales), the most abundant ones, were affected by all molecules except C16-HSL, and the four AHLs induced a similar response (mean log2FC = −0.70 and –2.60, respectively).

Similar to the First experiment, only a few ASVs were differentially abundant with the AHL treatment (22 out of 450 ASVs), but 12 of them (54%) (ASV 379, 400, 409, 512, 1,220, 1,221, 1,312, 1,325, 1,448, 1,450, 1,452, and 1,457) belonged to the 50 most abundant ASVs present in the DMSO control at the end of the incubation (Supplementary Table 1), representing 13.4% of the control community.

### Associations Between Specific Enzymatic Activities and Diversity

For both experiments, Procrustes analysis revealed significant associations between the specific enzymatic activities assayed at 48 h and the final diversity (First experiment: *m*^2^ = 0.54, *r* = 0.68, *p* = 0.001; Second experiment: *m*^2^ = 0.59, *r* = 0.64, *p* = 0.001) (Supplementary Figure 6).

## Discussion

Marine microorganisms, especially heterotrophic prokaryotes, play a major role in marine biogeochemical cycles by consuming and recycling organic matter and nutrients. Their recycling capacities depend, among others, on the community composition and the differential expression of this genomic potential, which could be impacted by cell-cell communication systems such as AHL-based QS. Our current knowledge of QS mechanisms mostly stems from isolated bacteria cultivated *in vitro*, which established the basic molecular foundation of QS communication (Lami, 2019). However, natural bacterial communities consist of complex assemblages facing fluctuating environmental conditions. As such, it is crucial to understand the role of QS communications in these communities (Hmelo, 2017; Mukherjee and Bassler, 2019). A few studies have examined the impact of AHL amendment on hydrolytic enzyme activities from marine snow-attached bacteria (Hmelo et al., 2011; Van Mooy et al., 2012; Jatt et al., 2015; Krupke et al., 2016; Su et al., 2019). However, to the best of our knowledge, none were conducted on planktonic bacterial communities. In addition, the effect of AHL amendments on the composition of marine bacterial assemblages is currently unknown. To bridge this gap, this study examined the variability in the effect of AHL amendments on several hydrolases and the BCC of planktonic bacterial communities sampled in coastal waters (Bay of Brest, France).

### *N*-Acylhomoserine Lactones as a Nutritional Source

As first suggested by Hmelo et al. (2011), the impact of the AHL amendments could result from their use as a C and/or N source, instead of a QS-based regulation. However, similar to previous studies (Hmelo et al., 2011; Krupke et al., 2016), we discarded this hypothesis for several reasons. First, the microcosms were amended with 50 nM of AHL, equaling between 0.4 and 1 μM of C (depending on the AHL side-chain length), which is well beneath the usual dissolved organic C concentration in the studied waters (around 70 μM at this time of the year) (Dulaquais et al., 2018). Furthermore, the amendments did not affect the bacterial abundance compared to the control condition, suggesting that AHLs are not an additional nutritional source. In addition, different AHLs elicited both increase and decrease in specific hydrolytic activities and acted on a limited number of activities (which differed between each experiment). Those two points suggest a specific regulation of particular phenotypes as opposed to a global increase in bacterial metabolism. Finally, in the preliminary studies used to determine the AHL concentration (Supplementary Figure 2), the impact of AHL seemed to lessen with increased molecule concentration, which is counterintuitive with their use as a nutritional source. As such, it is likely that the observed changes in specific activities and BCC do not result from the use of AHL as C and/or N source.

### Quorum Sensing Modulates the Hydrolytic Activity of Marine Bacterial Communities

To investigate the involvement of QS in hydrolases production, we monitored several enzymatic activities involved in the degradation of different types of molecules (polysaccharides, peptides, and lipids) and elements (C, N, and P) throughout the microcosm duration: β-glc, N-ac, and β-glucu activities are related to polysaccharides cycling; Lip and LAM activities, respectively, degrade lipids and peptides while AP activities are involved in P cycling. Overall, our results highlighted the involvement of QS in hydrolytic enzyme regulation among natural marine bacterial communities. During the First experiment (early bloom), AHL addition mostly increased the specific N-ac and Lip activities, with C6-HSL being the most potent AHL. In contrast, at the beginning of the Second experiment (peak bloom), the specific LAM activity decreased and the specific β-glucu activities increased with all added AHLs, especially C12- and 3-oxo-C8-HSL. Enzymatic activities were most largely impacted after 6 h of incubation, suggesting that their synthesis was driven by a transcriptional QS-based mechanism. Indeed, a modification of activities driven by changes in BCC is more likely to occur over a longer timescale (>24 h).

Within the same experiment, the different AHLs mostly induced similar responses, which is in contrast with the results reported by Krupke et al. (2016), where two AHLs rarely elicited the same response. However, the effect of AHL was highly variable from the First to Second experiment: none of the tested activities responded similarly between the two experiments. This variability in the response to AHL amendment is not surprising given the complexity of the mechanisms at play. The response of the bacterial community to AHL amendment may depend, for instance, on the initial BCC (presence/absence of QS and QQ genes), which differed between the two experiments (Supplementary Figures 7, 8). Different AHL degradation capacities, mediated for instance by AHL-lactonases or AHL-acylases, could especially influence the fate of the amended AHLs. In addition, global gene regulation results from the assimilation of different signals (Nickzad and Déziel, 2016; Rolland et al., 2016; Whiteley et al., 2017; Mukherjee and Bassler, 2019). In particular, AHL-based QS regulations are modulated by signaling molecules (including AHLs and other AI types) (Long et al., 2009), C sources (Medina-Martínez et al., 2006; Shrout et al., 2006), nutrient levels (McIntosh et al., 2009; Boyle et al., 2015), or algal compounds (Rolland et al., 2016; Dow, 2021 and ref. therein), among others. The initial bacterial communities were sampled at the beginning and the peak of the spring phytoplankton growth (Supplementary Figure 1), which is likely to have a major impact on those parameters. The variability of responses between the two experiments is in concordance with previously reported results. For instance, Krupke et al. (2016) noted that the effect of AHL amendment on hydrolytic activity was “remarkably inconsistent” when comparing several sampling locations.

Taken together, the AHL amendment experiments from this study and from previous ones demonstrate the involvement of QS-based communication in hydrolytic activity regulation. They also highlight the complexity of such regulation as no pattern seems to emerge from the different AHL amendment experiments.

### Quorum Sensing Modulates the Composition of Marine Bacterial Communities

We then investigated whether AHL amendment modulated the BCC by monitoring the diversity, using metabarcoding of the V3/V4 region of the 16S rRNA gene. Both the PCoA ordination (and subsequent PERMANOVA and ANOSIM tests) and the differential abundance analyses revealed a significant effect of the AHL treatment on the BCC compared to the DMSO control. In addition, although few ASVs were affected, they were amongst the most abundant ASVs in the control community for both experiments. During the First experiment, C6-HSL and 3-oxo-C8-HSL affected the largest number of ASVs, including Bacteroidia and Gammaproteobacteria. The other AHLs mainly affected Bacteroidia. During the Second experiment, the effect of the AHL treatment seemed to differ according to the side-chain length of the AHL: the short-chain AHLs (C4-, C6-, and 3-oxo-C8-HSL) reduced the abundance of a core of two Flavobacteriales ASVs. The C12-HSL affected the largest number of ASVs, including the same two Flavobacteriales ASVs as well as several Gammaproteobacteria. In contrast, the C16-HSL mostly affected Gammaproteobacteria.

Overall, the affected ASVs were affiliated to Bacteroidia (only Flavobacteriales and mostly Flavobacteriaceae), Gammaproteobacteria (mainly Vibrionales, Alteromonadales, and Oceanospirillales) and Alphaproteobacteria (only Rhodobacterales), which is in agreement with the literature. Indeed, the members of Gamma- and Alphaproteobacteria are broadly involved in QS communications. Several environmental Vibrionales, Alteromodales, and Rhodobacterales strains use AHL-based QS (Gram et al., 2002; Cuadrado-silva et al., 2013; Su et al., 2019). In addition, several marine metagenomic datasets have shown the important contribution of Rhodobacterales, Vibrionales, Alteromonadales, Cellvibrionales, and Oceanospirillales to the number of sequences coding for AHL synthases (Doberva et al., 2015; Huang et al., 2018; Su et al., 2021), AHLs receptors (Su et al., 2021) or AHL QQ genes (Huang et al., 2018; Su et al., 2021). Similarly, several cultivated Flavobacteriaceae species can produce or degrade AHLs during cultivation (Romero et al., 2010; Su et al., 2019). In addition, Flavobacteriales have been found to code for AHL receptors and AHL QQ genes (Su et al., 2021) in marine metagenomic datasets.

To our knowledge, no study has previously demonstrated the direct effect of AHL amendment on marine planktonic bacterial community structuration. However, a few studies have suggested that QS-based cell-cell communications may be an overlooked mechanism structuring marine bacterial communities. For instance, Whalen et al. (2019) showed that 2-heptyl-4-quinolone (HHQ, another type of QS signal belonging to the alkylquinolone) amendment to microbial communities sampled along a simulated bloom differently modulated the BCC depending on the bloom stage. The authors found, among others, that HHQ amendment increased the relative abundances of Gammaproteobacteria but decreased those of Bacteroidetes. This seems to be in agreement with our study, where two distinct communities sampled at different stages of the spring phytoplankton growth responded differently to AHL amendment. In addition, Huang et al. (2019) demonstrated that QS disruption through lactonase-based degradation of AHLs impacted the diversity of colonized immerged steel coupons, reducing the abundances of Burkholderiales, Pseudomonadales, and Rhodospirillales, among others.

It is mostly unclear how QS affected BCC, which probably resulted from the combined action of several mechanisms. First, QS can regulate the production of “public goods” (Whiteley et al., 2017; Mukherjee and Bassler, 2019), which are common-pool compounds or functions providing a collective benefit (Smith and Schuster, 2019). They include, among others, extracellular hydrolases, siderophores, or biosurfactants. The upregulation or downregulation of those common goods would likely drive changes in BCC. For instance, the evidenced modification in hydrolytic activities could allow non-degrading “cheater” cells to grow. Interestingly, in our study, the effect of AHL treatment on the BCC seemed to mirror the effect on specific enzymatic activities, as suggested by the Procrustes analysis, which supports this hypothesis. Second, QS-regulated mechanisms could impact microbial communities through the production of antimicrobial compounds that kill or inhibit the growth of microorganisms (including antibiotics or algaecides), which has previously been documented in aquatic strains (Rolland et al., 2016; Mion et al., 2021). Finally, it is possible that AHL-driven changes in BCC resulted from the additional functions of AHLs (or their degradation products), which include antibacterial activity and iron chelation properties (Kaufmann et al., 2005; Schertzer et al., 2009).

### Relevance of Quorum Sensing Communications *in situ*

In marine and aquatic environments, QS is believed to occur in biofilm-associated communities, covering biotic (such as phytoplankton or coral) or abiotic surfaces (such as marine snow) (Hmelo and Van Mooy, 2009; Hmelo, 2017). Those densely populated would allow the AHL induction threshold to be reached, whereas this seems unrealistic in free-living, open-ocean communities. However, the quick phenotypic response observed in our experiments (between 0 and 6 h) suggests that QS has implications beyond those biofilm environments. Indeed, the diffusion of AHLs in the vicinity of biofilms, for example in marine snow plume or in the phycosphere (Stocker, 2012), can potentially impact the surrounding free-living communities on such a short time-scale. The modulation of the free-living community genes’ expression may then drive changes in its composition and functions. Such interpretation has been proposed by Schwab et al. (2019), who demonstrated that AHL disruption in soil communities affected not only the biofilm formation, but also the suspended communities, suggesting that AHL signaling extends beyond biofilms.

## Conclusion

Overall, this study demonstrates that AHL-based QS is involved in regulating hydrolytic enzyme production and BCC structuration in marine bacterial communities, which drive mineralization pathways. If initial experiments carried out on marine snow suggested that particulate organic matter degradation is coordinated by QS, this study extended those results to free-living bacterial coastal communities for the first time. In addition, to the best of our knowledge, this study is the first to demonstrate the direct impact of AHL amendment on the composition of marine planktonic bacterial communities. As such, QS communication pathways could be an unaccounted mechanism with profound ecological and biogeochemical implications for the oceans. The variability of response following AHL amendment points out the complexity of cell-cell communication mechanisms. In the future, more research will be needed to better characterize the function and the mechanisms of action of bacterial cell-cell communications in free-living and surface-attached communities, using both isolated strains, synthetic microbial communities, and natural assemblages.

## Data Availability Statement

The datasets presented in this study can be found in online repositories. The names of the repository/repositories and accession number(s) can be found below: https://www.ncbi.nlm.nih.gov/search/all/?term=PRJNA759568.

## Author Contributions

MU, CL, and DD performed the experiments. MU analyzed the data and wrote the first draft of the manuscript. All authors contributed to the conception and design of the study, manuscript revision, read, and approved the submitted version.

## Conflict of Interest

The authors declare that the research was conducted in the absence of any commercial or financial relationships that could be construed as a potential conflict of interest.

## Publisher’s Note

All claims expressed in this article are solely those of the authors and do not necessarily represent those of their affiliated organizations, or those of the publisher, the editors and the reviewers. Any product that may be evaluated in this article, or claim that may be made by its manufacturer, is not guaranteed or endorsed by the publisher.

## Footnotes

1
http://somlit.fr

2
https://github.com/ifremer-bioinformatics/samba
