# Supplementary material for: Quorum Sensing Regulates the Hydrolytic Enzyme Production and Community Composition of Heterotrophic Bacteria in Coastal Waters
Source: Front Microbiol. 2021 Dec 10;12:780759. doi: 10.3389/fmicb.2021.780759 (PMC8709541; doi:10.3389/fmicb.2021.780759)
Supplement: Supplementary file 1 [file Table_1.DOCX]

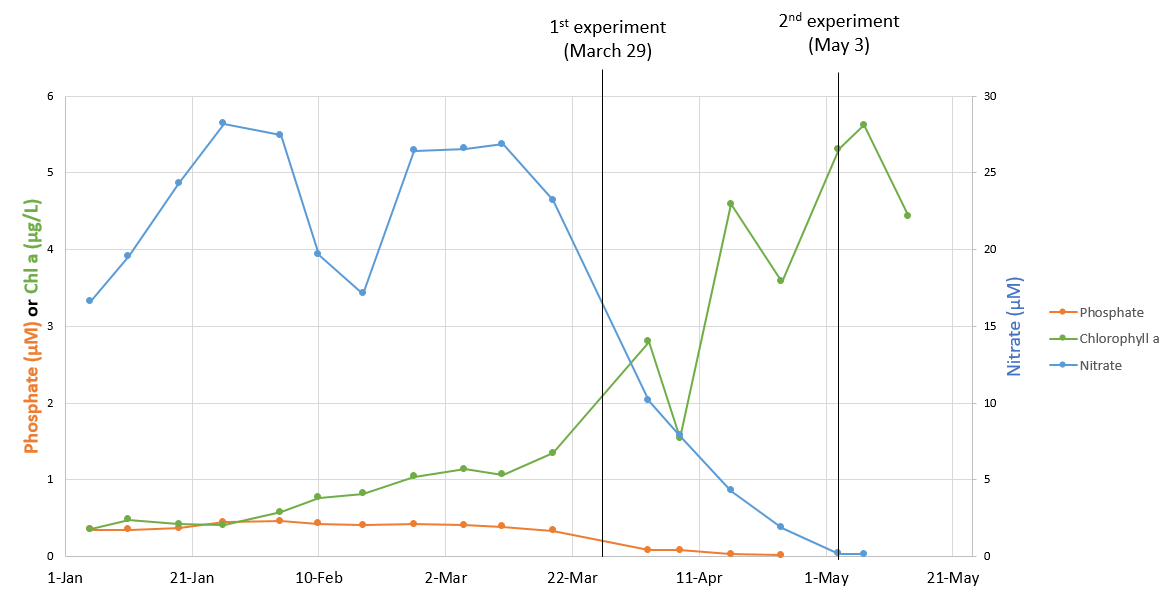


**Figure S1**: Environmental parameters (nitrate, phosphate and chlorophyll *a*) on the two experiment sampling dates, corresponding to the beginning (March 29) and the peak (May 3) of the phytoplankton spring growth. Data were downloaded from the SOMLIT website (https://www.somlit.fr/visualisation-des-donnees/) on September 17^th^ 2021.

The phytoplankton communities were identified by the observation network SNO PHYTOBS (Service National d’Observation du Phytoplancton, https://www.phytobs.fr/) on dates close to the experiments (March 26 and May 2, 2021). On March 26, close to the 1^st^ experiment (early bloom), the phytoplankton community was characterized by the presence of diatoms including *Pseudo-nitzschia* (16% of total cells), *Chaetoceros* (12.5%) and unidentified centric diatoms (12.5%). The nanophytoplankton included *Cryptophyceae* (27%). On May 2, near the 2^nd^ experiment (peak bloom), the phytoplankton community was different and mostly dominated by the diatom *Guinardia flaccida* (10%). The nanophytoplankton community included *Cryptophyceae* (57%) and *Choanoflagellates* (15%).

***Choice of AHL concentration***

A preliminary experiment was performed to determine which AHLs concentration induced the most important response in three hydrolytic activities (leucine-aminopeptidases, alkaline phosphatases, β-glucosidases). To this extent, brackish water was sampled in the Aulne estuary (Bay of Brest, France) on June 9, 2019 and filtered using a 10-µm silk (Merck, Ref NY1004700). Then, C6- or C14-HSL were amended at 50, 200, 500, 1000 or 5000 nM with a constant final DMSO concentration (0.1%). Incubation were carried out at *in situ* temperature (22°C) and the resulting activity was assessed at 48 h. Hydrolytic enzymes were assayed as described in the main script, in technical duplicates.

Although no statistical test was performed given the absence of biological replicates, alkaline phosphates seemed induced with 50 nM of C14-HSL (4.4 times increase, **Fig. S2**). The C14-HSL effect seemed to lessen with increasing AHL concentration (1.6-2.2 times increase with 200 to 5000 nM, Fig. S1). The C6-HSL also seemed to induce the alkaline phosphatase, especially using 50 nM of C6-HSL but no clear trend was visible among the different concentrations (**Fig. 2**). There was no clear pattern of induction for leucine aminopeptidases and β-glucosidases (not shown). Consequently, we choose to perform the experiments using 50 nM of AHLs.


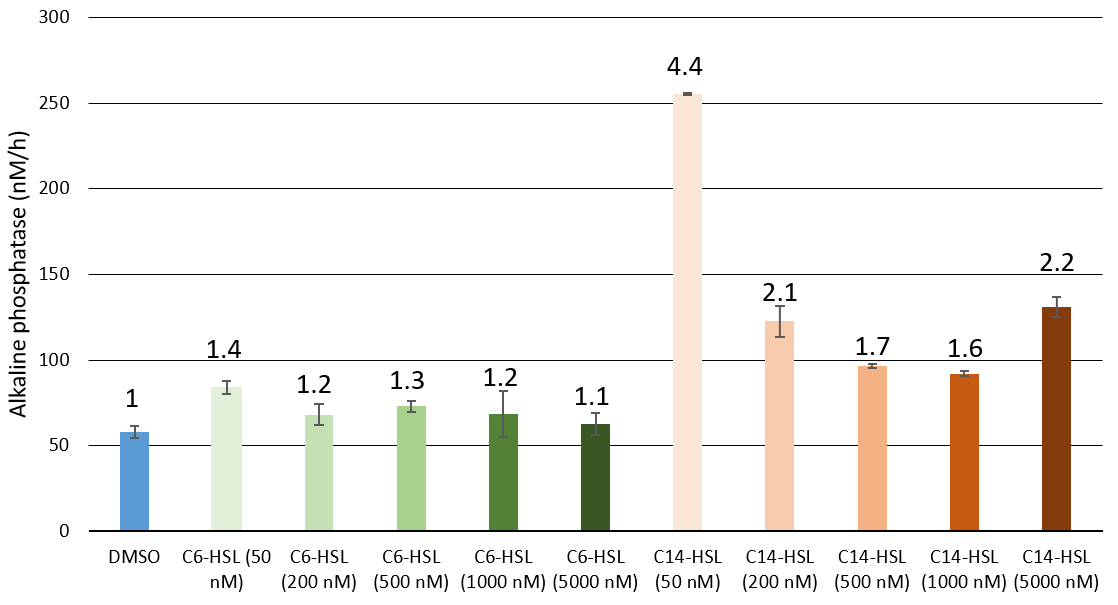


**Fig. S2:** Alkaline phosphatase 48 h after C6- and C14-HSL amendments. The error bars indicate standard error over technical duplicates. The ratio between the treatment and the DMSO control is annotated above the error bars.


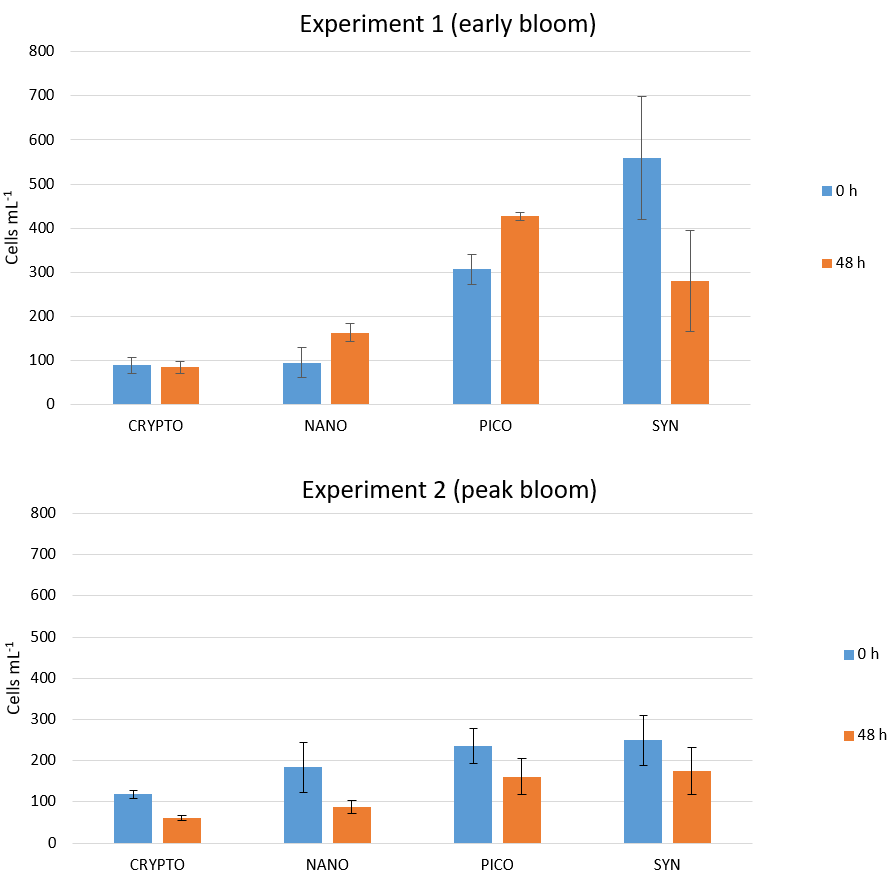


**Fig. S3:** Quantification of the phytoplankton cells in the 10-µm filtered seawater before dispatching (blue) and in the DMSO control at the end of the 48 h incubation (orange) for the 1st (early bloom, top) and the 2nd experiment (peak bloom, bottom). CRYPTO: cryptophytes, NANO: nanophytoplankton, PICO: picophytoplankton, SYN: *synechococcus*.


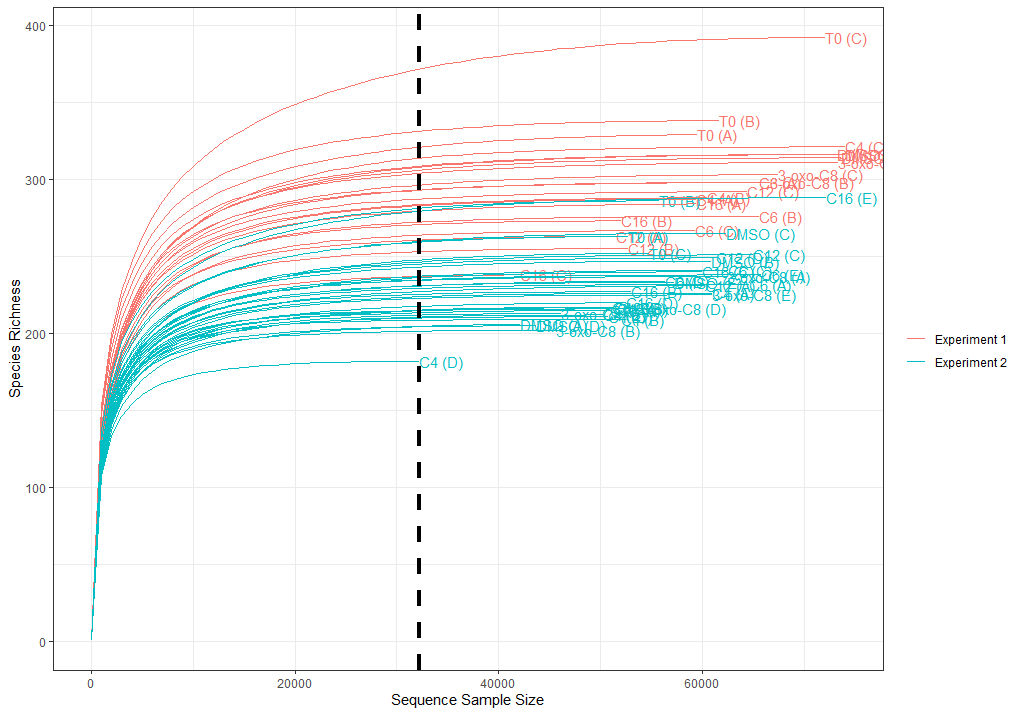


**Fig. S4:** Rarefaction curves for the two experiments. The vertical dashed bar represent the number of reads used for rarefaction (32 196 sequences per sample).

**A**)


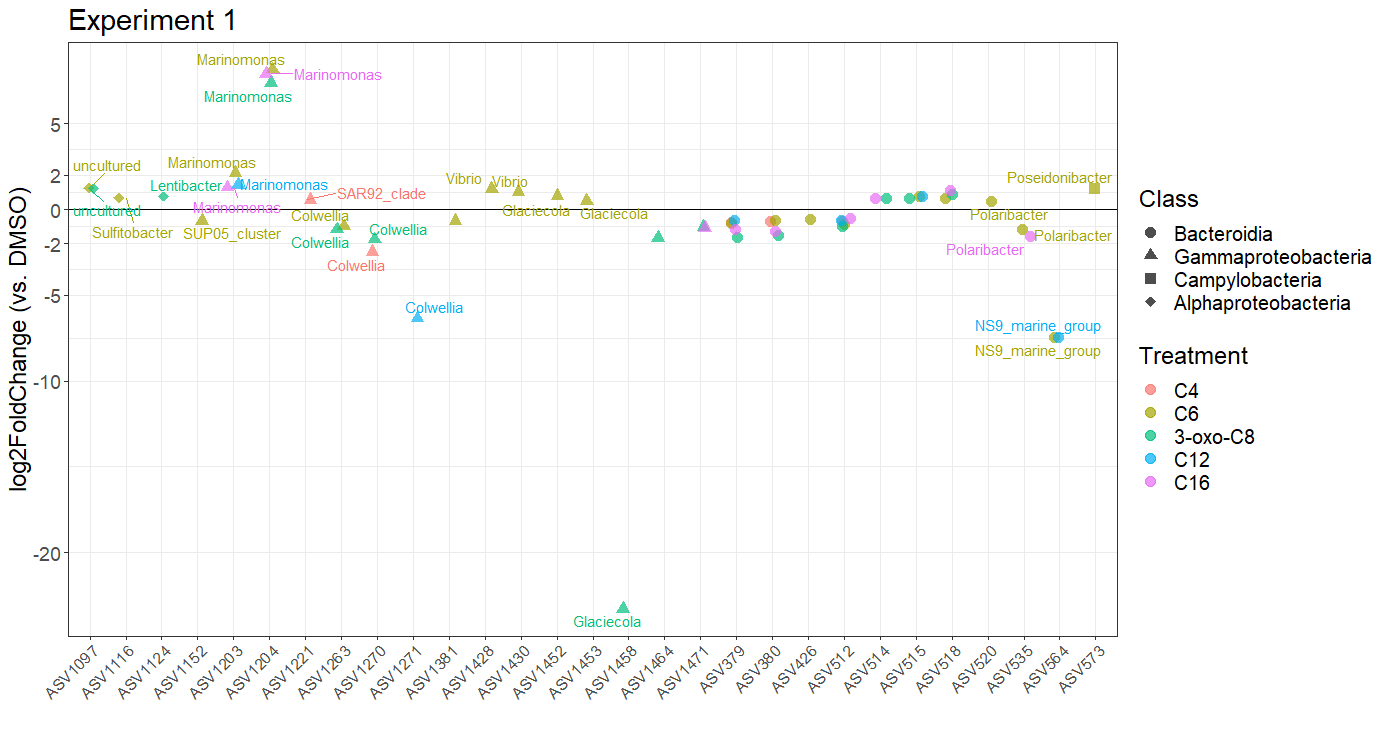


**B**)


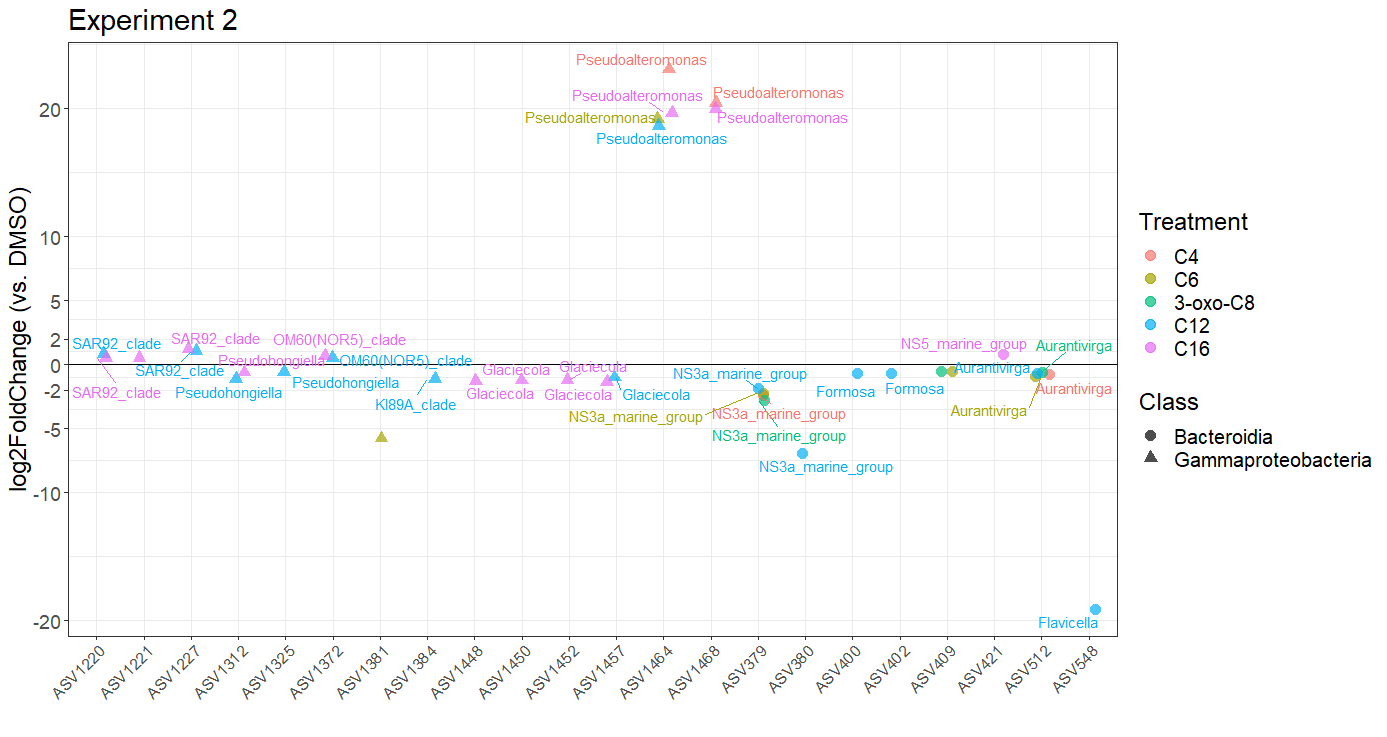


**Fig. S5:** Log2 fold change of the differentially abundant ASVs for each AHL-treatment compared to the DMSO control (cut-off p_BH_ = 0.05, cut-off log2FC = 0.5 or -0.5) for the 1^st^ (early bloom, **A**) and 2^nd^ (peak bloom) experiment (**B**)

**A)**

**
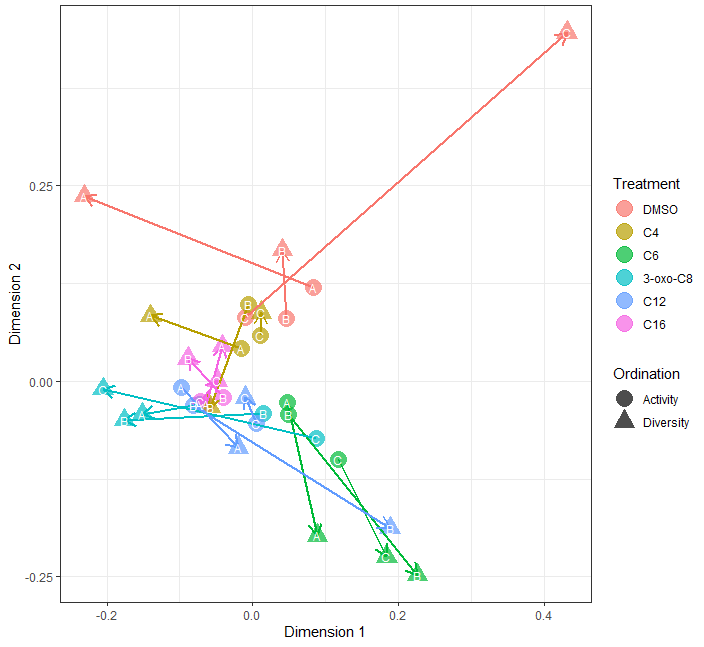
**

**B)**


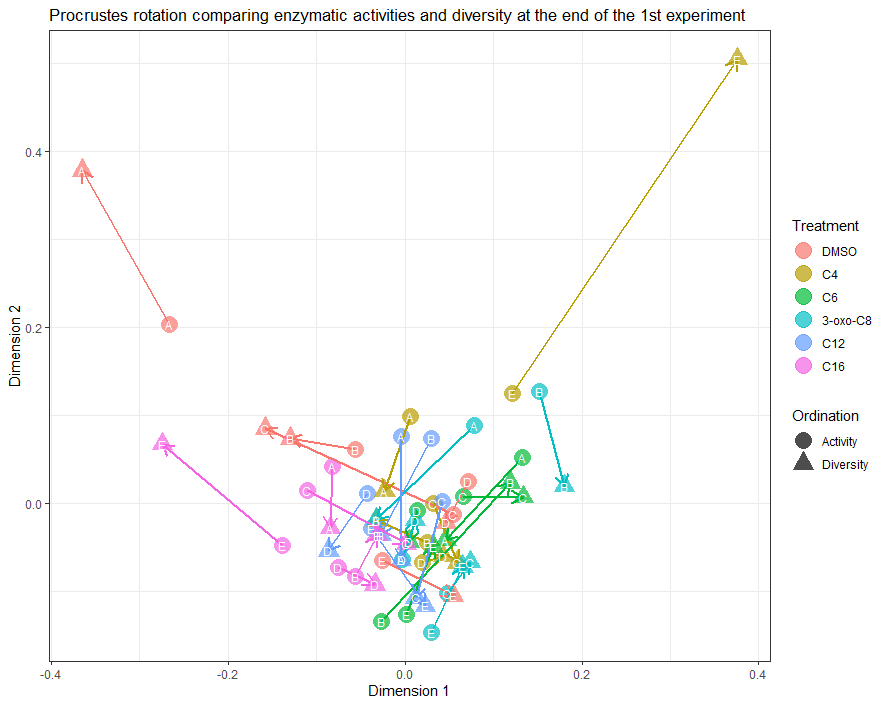


**Fig. S6.** Procrustes plot showing the superimposition of the specific activities and the BCC ordinations for the 1^st^ experiment (early bloom, **A**) and the 2^nd^ experiment (peak bloom, **B**).


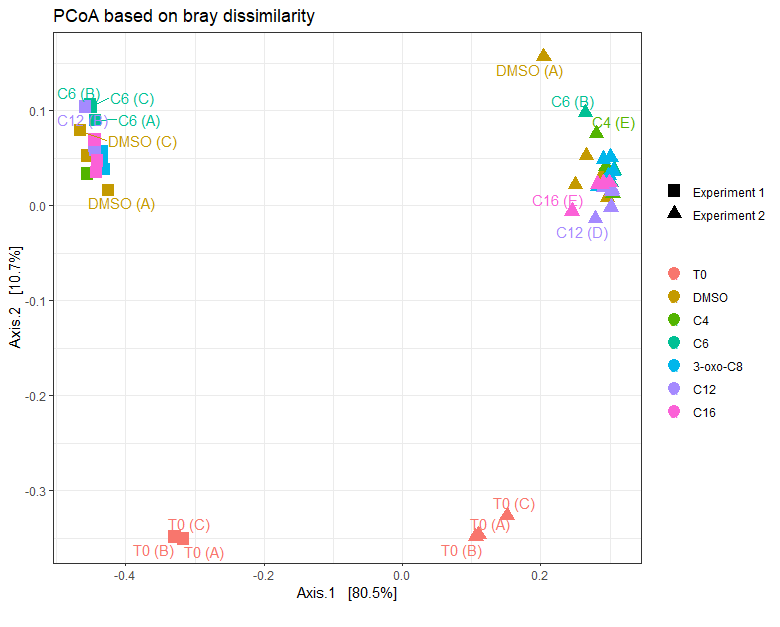


**Fig. S7:** Principal Coordinates analysis (PCoA) based on the Bray-Curtis dissimilarity for both experiments.


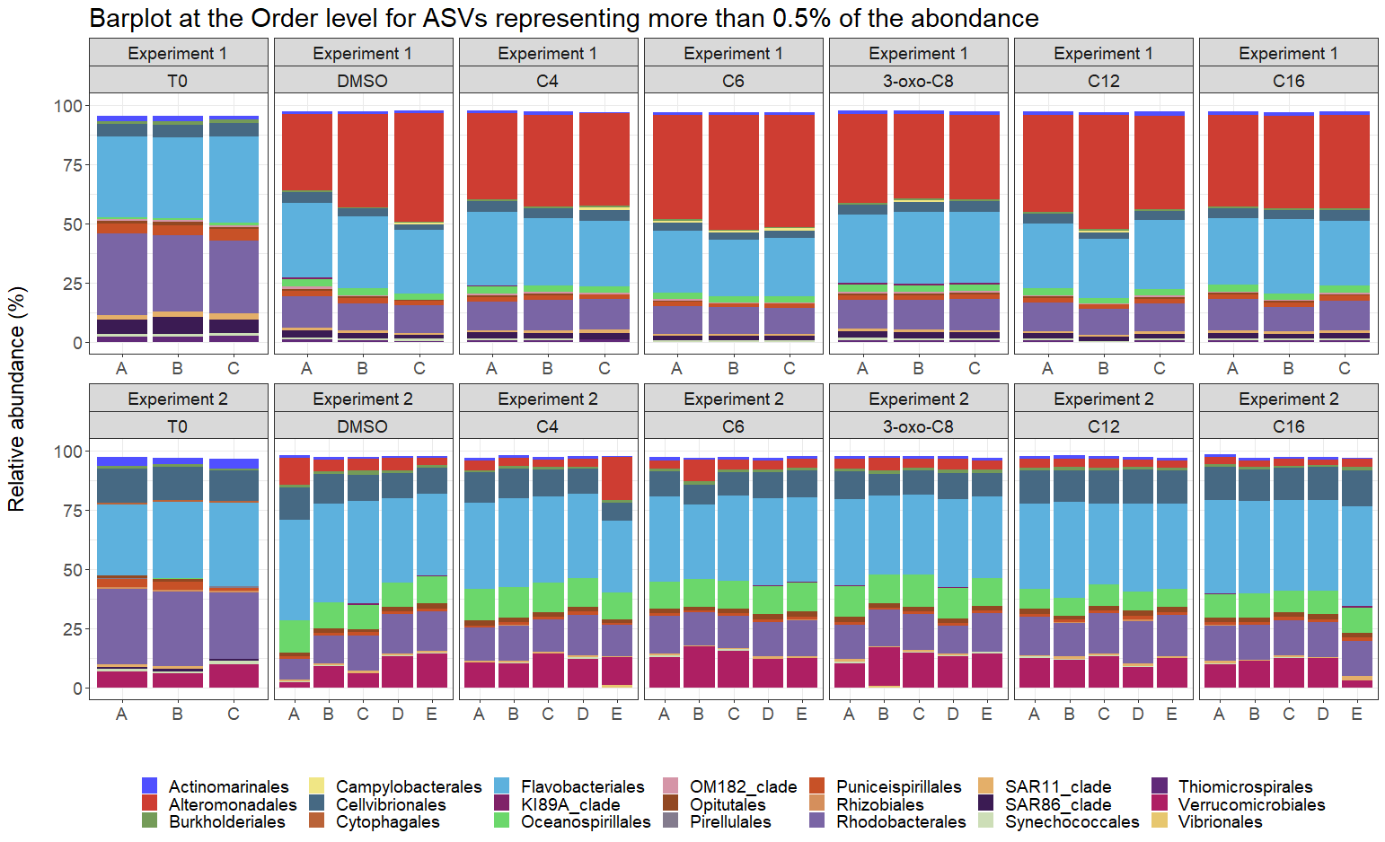


**Fig. S8**: Composition of the communities in the 1^st^ (early bloom, top panels) and 2^nd^ experiment (peak bloom, bottom panels) at the beginning (T0) and end of the 48h incubation, for the different treatments (DMSO, C4-HSL, C6-HSL, 3-oxo-C8-HSL, C12-HSL and C16-HSL). Only the ASVs representing more than 0.5% of the sample abundance are represented, agglomerated at the Order level. The different replicates are labeled A, B, C, D and E in the x-axis.

**Table S1:** Mean relative abundance of the 50 most abundant ASVs in the DMSO control at the end of the 48 h incubation, for the 1^st^ (early bloom, left) and 2^nd^ (peak bloom, right) experiment. Mean relative abundance (Ab) and standard deviation (SD) were calculated from the biological replicates. The differentially abundant ASVs were highlighted in orange (differentially abundant in one experiment) or green (differentially abundant in both experiments).

| **Experiment 1** | | | | | **Experiment 2** | | | | | |
| --- | --- | --- | --- | --- | --- | --- | --- | --- | --- | --- |
| **ASV** | **Order** | **Genus** | **Ab (%)** | **SD** | **ASV** | **Order** | **Genus** | **Ab (%)** | **SD** |  |
| **ASV1457** | Alteromonadales | Glaciecola | 22.8 | 4.71 | **ASV537** | Flavobacteriales | Flavicella | 11.9 | 1.15 |  |
| **ASV520** | Flavobacteriales | Polaribacter | 7.4 | 2.31 | **ASV396** | Flavobacteriales | Winogradskyella | 10.7 | 2.78 |  |
| **ASV1450** | Alteromonadales | Glaciecola | 6.71 | 0.75 | **ASV708** | Verrucomicrobiales | Persicirhabdus | 8.11 | 4.98 |  |
| **ASV1073** | Rhodobacterales | Amylibacter | 6.45 | 0.91 | **ASV1133** | Rhodobacterales | Planktomarina | 7.76 | 3.32 |  |
| **ASV512** | Flavobacteriales | Aurantivirga | 5.66 | 0.81 | **ASV1329** | Oceanospirillales | Pseudohongiella | 7.48 | 0.89 |  |
| **ASV1452** | Alteromonadales | Glaciecola | 4.43 | 0.71 | **ASV1219** | Cellvibrionales | SAR92_clade | 3.66 | 1.03 |  |
| **ASV1133** | Rhodobacterales | Planktomarina | 4.17 | 1.37 | **ASV494** | Flavobacteriales | uncultured | 2.77 | 0.95 |  |
| **ASV426** | Flavobacteriales | NS5_marine_group | 3.81 | 0.66 | **ASV1312** | Oceanospirillales | Pseudohongiella | 2.75 | 0.42 |  |
| **ASV1221** | Cellvibrionales | SAR92_clade | 1.95 | 0.79 | **ASV512** | Flavobacteriales | Aurantivirga | 2.66 | 0.78 |  |
| **ASV1312** | Oceanospirillales | Pseudohongiella | 1.41 | 0.11 | **ASV1105** | Rhodobacterales | Ascidiaceihabitans | 2.36 | 0.33 |  |
| **ASV1453** | Alteromonadales | Glaciecola | 1.08 | 0.11 | **ASV1214** | Cellvibrionales | SAR92_clade | 2.12 | 0.31 |  |
| **ASV1351** | SAR86_clade | SAR86_clade | 1.02 | 0.31 | **ASV1073** | Rhodobacterales | Amylibacter | 2.11 | 0.16 |  |
| **ASV1052** | Puniceispirillales | Candidatus_Puniceispirillum | 1 | 0.14 | **ASV1450** | Alteromonadales | Glaciecola | 1.89 | 1.2 |  |
| **ASV389** | Flavobacteriales | NS2b_marine_group | 0.93 | 0.16 | **ASV1226** | Cellvibrionales | SAR92_clade | 1.6 | 0.06 |  |
| **ASV409** | Flavobacteriales | NS5_marine_group | 0.91 | 0.06 | **ASV1457** | Alteromonadales | Glaciecola | 1.59 | 1.09 |  |
| **ASV773** | Actinomarinales | Candidatus_Actinomarina | 0.87 | 0.04 | **ASV1220** | Cellvibrionales | SAR92_clade | 1.45 | 0.2 |  |
| **ASV513** | Flavobacteriales | Aurantivirga | 0.85 | 0.09 | **ASV383** | Flavobacteriales | Ulvibacter | 1.28 | 0.26 |  |
| **ASV436** | Flavobacteriales | NS4_marine_group | 0.81 | 0.23 | **ASV518** | Flavobacteriales | Polaribacter | 1.16 | 0.33 |  |
| **ASV1059** | Puniceispirillales | SAR116_clade | 0.79 | 0.02 | **ASV471** | Flavobacteriales | Fluviicola | 1 | 0.1 |  |
| **ASV518** | Flavobacteriales | Polaribacter | 0.74 | 0.33 | **ASV405** | Flavobacteriales | Formosa | 0.83 | 0.14 |  |
| **ASV496** | Flavobacteriales | uncultured | 0.73 | 0.1 | **ASV773** | Actinomarinales | Candidatus_Actinomarina | 0.8 | 0.16 |  |
| **ASV494** | Flavobacteriales | uncultured | 0.72 | 0.07 | **ASV409** | Flavobacteriales | NS5_marine_group | 0.79 | 0.14 |  |
| **ASV1372** | Cellvibrionales | OM60(NOR5)_clade | 0.68 | 0.19 | **ASV699** | Verrucomicrobiales | Roseibacillus | 0.7 | 0.11 |  |
| **ASV259** | Synechococcales | Synechococcus_CC9902 | 0.62 | 0.07 | **ASV1448** | Alteromonadales | Glaciecola | 0.63 | 0.47 |  |
| **ASV379** | Flavobacteriales | NS3a_marine_group | 0.61 | 0.08 | **ASV1275** | Alteromonadales | Colwellia | 0.61 | 0.2 |  |
| **ASV515** | Flavobacteriales | Polaribacter | 0.6 | 0.26 | **ASV1400** | Burkholderiales | OM43_clade | 0.61 | 0.1 |  |
| **ASV1240** | Alteromonadales | Colwellia | 0.53 | 0.2 | **ASV538** | Flavobacteriales | Flavicella | 0.61 | 0.18 |  |
| **ASV1357** | OM182_clade | OM182_clade | 0.52 | 0.18 | **ASV677** | Opitutales | MB11C04_marine_group | 0.59 | 0.11 |  |
| **ASV1350** | SAR86_clade | SAR86_clade | 0.48 | 0.19 | **ASV514** | Flavobacteriales | Polaribacter | 0.58 | 0.15 |  |
| **ASV1325** | Oceanospirillales | Pseudohongiella | 0.47 | 0.03 | **ASV1124** | Rhodobacterales | Lentibacter | 0.54 | 0.12 |  |
| **ASV1454** | Alteromonadales | Glaciecola | 0.42 | 0.1 | **ASV1052** | Puniceispirillales | Candidatus_Puniceispirillum | 0.5 | 0.04 |  |
| **ASV1455** | Alteromonadales | Glaciecola | 0.42 | 0.1 | **ASV1213** | Cellvibrionales | SAR92_clade | 0.5 | 0.05 |  |
| **ASV1332** | Oceanospirillales | Pseudohongiella | 0.4 | 0.08 | **ASV400** | Flavobacteriales | Formosa | 0.5 | 0.11 |  |
| **ASV1105** | Rhodobacterales | Ascidiaceihabitans | 0.39 | 0.07 | **ASV978** | SAR11_clade | Clade_Ia | 0.47 | 0.22 |  |
| **ASV1152** | Thiomicrospirales | SUP05_cluster | 0.39 | 0.14 | **ASV1059** | Puniceispirillales | SAR116_clade | 0.44 | 0.04 |  |
| **ASV514** | Flavobacteriales | Polaribacter | 0.39 | 0.11 | **ASV497** | Flavobacteriales | uncultured | 0.43 | 0.06 |  |
| **ASV424** | Flavobacteriales | NS5_marine_group | 0.38 | 0.09 | **ASV663** | Opitutales | MB11C04_marine_group | 0.4 | 0.08 |  |
| **ASV386** | Flavobacteriales | uncultured | 0.37 | 0.07 | **ASV416** | Flavobacteriales | NS5_marine_group | 0.37 | 0.07 |  |
| **ASV975** | SAR11_clade | Clade_Ia | 0.36 | 0.09 | **ASV569** | Flavobacteriales | NS9_marine_group | 0.37 | 0.1 |  |
| **ASV978** | SAR11_clade | Clade_Ia | 0.36 | 0.11 | **ASV259** | Synechococcales | Synechococcus_CC9902 | 0.33 | 0.08 |  |
| **ASV1252** | Alteromonadales | Colwellia | 0.35 | 0.29 | **ASV1330** | Oceanospirillales | Pseudohongiella | 0.32 | 0.06 |  |
| **ASV1124** | Rhodobacterales | Lentibacter | 0.34 | 0.11 | **ASV379** | Flavobacteriales | NS3a_marine_group | 0.32 | 0.03 |  |
| **ASV1382** | KI89A_clade | KI89A_clade | 0.33 | 0.16 | **ASV1397** | Burkholderiales | OM43_clade | 0.3 | 0.09 |  |
| **ASV410** | Flavobacteriales | NS5_marine_group | 0.31 | 0.02 | **ASV669** | Opitutales | MB11C04_marine_group | 0.3 | 0.05 |  |
| **ASV380** | Flavobacteriales | NS3a_marine_group | 0.3 | 0 | **ASV1382** | KI89A_clade | KI89A_clade | 0.29 | 0.12 |  |
| **ASV1346** | SAR86_clade | SAR86_clade | 0.29 | 0.07 | **ASV1452** | Alteromonadales | Glaciecola | 0.28 | 0.22 |  |
| **ASV578** | Campylobacterales | uncultured | 0.28 | 0.14 | **ASV520** | Flavobacteriales | Polaribacter | 0.28 | 0.03 |  |
| **ASV498** | Flavobacteriales | uncultured | 0.27 | 0.03 | **ASV1325** | Oceanospirillales | Pseudohongiella | 0.26 | 0.02 |  |
| **ASV1459** | Alteromonadales | Glaciecola | 0.26 | 0.23 | **ASV1399** | Burkholderiales | OM43_clade | 0.26 | 0.02 |  |
| **ASV771** | Actinomarinales | Candidatus_Actinomarina | 0.25 | 0.04 | **ASV1221** | Cellvibrionales | SAR92_clade | 0.24 | 0.08 |  |
